# Supplementary material for: Kinetics of cytokine receptor trafficking determine signaling and functional selectivity
Source: eLife. 2019 Nov 27;8:e49314. doi: 10.7554/eLife.49314 (PMC6914340; doi:10.7554/eLife.49314)
Supplement: Supplementary file 1. [file elife-49314-supp1.pdf]

Gene Name	Transcript Length (base pairs, bp)	Number of STAT3 binding sites	Location of STAT3 binding
**SOCS3**	3,300	4	promoter and down-stream enhancer
**BCL3**	11,498	4	promoter and intergenic
**SBNO2**	66,648	1	intergenic
**ZBED2**	110,364	1	up-stream enhancer
**MUC1**	7,092	1	promoter
**GZMB**	3,314	1	promoter
**PDCD1**	9,028	2	promoter and down-stream enhancer
**SLC19A1**	46,211	1	up-stream enhancer
**SREBF1**	24,930	1	up-stream enhancer
**BCL6**	24,351	1	promoter
**JAK3**	23,247	2	promoter and intergenic
**MXD1**	27,898	N/A	
**PIM2**	5,843	3	promoter
**SOCS1**	1,775	1	down-stream enhancer
**CMTM8**	131,647	1	intergenic
**PIM1**	5,224	1	Down-stream enhancer
**PARP9**	36,654	1	intergenic
**LDLR**	42,802	1	promoter
**IFI30**	4,451	N/A	
**CNP**	10,991	1	promoter
**IFITM1**	1,767	1	promoter
**ANK3**	705,090	1	promoter
**GIMAP5**	21,394	1	down-stream enhancer
**NELL2**	368,097	N/A	
